# Anticancer Activity of Natural Products and Related Compounds

**DOI:** 10.3390/ijms242216507

**Published:** 2023-11-20

**Authors:** Barbara De Filippis, Marialuigia Fantacuzzi, Alessandra Ammazzalorso

**Affiliations:** Department of Pharmacy, G. d’Annunzio University of Chieti-Pescara, Via dei Vestini 31, 66100 Chieti, Italy; barbara.defilippis@unich.it (B.D.F.); alessandra.ammazzalorso@unich.it (A.A.)

Nature has always been a precious source of bioactive molecules which are used for the treatment of various diseases [1]. Natural compounds such as dietary phytochemicals, nutritional herbs, and their constitutive bioactive agents possess a great variety of chemical scaffolds and distinct bioactivity profiles, which make them suitable for applications in therapy or as valuable lead compounds to obtain novel potent bioactive compounds [2]. Significant advances in natural source isolation and extraction techniques have led to the identification of novel compounds as useful starting points for the generation of optimized molecules with enhanced therapeutic potential via semi-synthetic or synthetic processes [3].

The application of natural products in the field of chemotherapy and chemoprevention is a valuable research topic, leading to the extensive use of plant-derived compounds as potent antitumor molecules [4]. In addition, marine-based pharmaceuticals have been extensively studied for their applications in the anticancer field, providing useful compounds such as cytarabine and trabectedin [5]. Alternative treatments in complement with traditional methods (radiotherapy, chemotherapy, and surgery) have been shown to be helpful and offer very reasonable alternatives to current medicines for cancer [6]. Much effort has also been directed towards the discovery of novel targets [7,8,9] in an attempt to obtain anticancer effects via multiple mechanisms, overcoming the resistance phenomena developed by most cancers.

Natural products effectively inhibit cell proliferation, regulate the cell cycle, and interfere with several tumorigenic signaling pathways [10,11]. The anticancer properties of polyphenols, found abundantly in plants, as flavonoids [12], terpenoids [13], and alkaloids [14], have been extensively reported [15]. However, important research efforts are necessary to fully understand the mechanisms of action of natural compounds by which these agents affect cell proliferation, differentiation, apoptosis, angiogenesis, and metastasis; in addition, there is a need to overcome major problems such as toxicity, poor selectivity, and unfavorable pharmacokinetics [16].

Currently, many plant-based antitumor drugs are in clinical use, such as taxanes, vinblastine, vincristine, and podophyllotoxin analogues. The combined use of phytochemicals like resveratrol, curcumin, and thymoquinone with other antitumor agents has shown significant success in preclinical studies, allowing enhanced efficacy and mitigation of side effects [17,18]. Emerging nanotechnology applications for anticancer drug formulations have been revolutionizing cancer therapy. Tissue-specific nanomedicines play a key role in advanced cancer diagnostic techniques by using liposomes, micelles, and nanoparticles as effective delivery vehicles [19]. Moreover, medicinal plant extracts have proven most effective in various cancers, paving the way for developing novel therapeutic strategies [20]. Many studies have been based on crude aqueous and ethanol extracts, with few explorations of their mechanisms [21].

In this Topic, 30 original articles and 3 reviews have been collected, with a particular focus on the isolation of bioactive compounds from natural sources, the mechanisms of action of anticancer compounds at the cellular level, and the application of active molecules against a panel of solid and hematological cancers, including melanoma, breast, lung, colorectal, prostate, bladder, and gastric cancer. Most of the analyzed compounds were from natural sources, whereas some semi-synthetic derivatives were also identified and discussed.

The most recent findings on the effects of extracts and their constituents in treating various cancers are discussed. Most works focus on the effect of water or ethanolic extracts from natural plants or fungi, such as *Viscum album* var. *coloratum, Drimia Maritima*, *Trichosanthes*, *Lupinus albus*, *Bryopsis plumosa*, *Elephantus scaber* L., *Paejangsan*, *Coix Seed*, *Lupinus albus*, *Ocimum sanctum* Linn., *Euphorbia fischeriana*, *Moldavian dragonhead*, *Streptomyces ardesiacus*, *Cichorium intybus*, and *Trichosanthes*.

The extracts and the isolated components have proven effective against breast, colorectal, lung, bladder, myeloma, and prostate cancer through several mechanisms, including decreased tumor cell viability, modulation of cytokines, secretion of chemokines, modulation of ROS, reduction of specific MMP subtypes, apoptosis, cell cycle inhibition, or by downregulating MAPK.

Table 1 schematically illustrates the content of this Topic, with all the contributions published in the six participating journals.

## Figures and Tables

**Table 1:** Original articles and reviews collected in the six journals participating in the Topic.

Title	Author	Journal	Year	DOI
Fermented Mangosteen (*Garcinia mangostana* L.) Supplementation in the Prevention of HPV-Induced Cervical Cancer: From Mechanisms to Clinical Outcomes	Kharaeva, Z.	*Cancers*	2022	https://doi.org/10.3390/cancers14194707
Scabertopin Derived from *Elephantopus scaber* L. Mediates Necroptosis by Inducing Reactive Oxygen Species Production in Bladder Cancer In Vitro	Gao, Y.	*Cancers*	2022	https://doi.org/10.3390/cancers14235976
Therapeutic Potential of Deflamin against Colorectal Cancer Development and Progression	Silva, S.	*Cancers*	2022	https://doi.org/10.3390/cancers14246182
Design, Synthesis and Biological Evaluation of Neocryptolepine Derivatives as Potential Anti-Gastric Cancer Agents	Ma, Y.	*IJMS*	2022	https://doi.org/10.3390/ijms231911924
FOXO1 Is a Key Mediator of Glucocorticoid-Induced Expression of Tristetraprolin in MDA-MB-231 Breast Cancer Cells	Jeon, D.	*IJMS*	2022	https://doi.org/10.3390/ijms232213673
New Angucycline Glycosides from a Marine-Derived Bacterium *Streptomyces ardesiacus*	Anh, C.	*IJMS*	2022	https://doi.org/10.3390/ijms232213779
Flavones, Flavonols, Lignans, and Caffeic Acid Derivatives from *Dracocephalum moldavica* and Their In Vitro Effects on Multiple Myeloma and Acute Myeloid Leukemia	Jöhrer, K.	*IJMS*	2022	https://doi.org/10.3390/ijms232214219
Anti-Cancer Effects of a New Herbal Medicine PSY by Inhibiting the STAT3 Signaling Pathway in Colorectal Cancer Cells and Its Phytochemical Analysis	Han, S.	*IJMS*	2022	https://doi.org/10.3390/ijms232314826
Combination Therapy of Curcumin and Disulfiram Synergistically Inhibits the Growth of B16-F10 Melanoma Cells by Inducing Oxidative Stress	Fontes, S.	*Biomolecules*	2022	https://doi.org/10.3390/biom12111600
Efficient Synthesis for Altering Side Chain Length on Cannabinoid Molecules and Their Effects in Chemotherapy and Chemotherapeutic Induced Neuropathic Pain	Raup-Konsavage, W.	*Biomolecules*	2022	https://doi.org/10.3390/biom12121869
Ent-Abietane Diterpenoids from *Euphorbia fischeriana* and Their Cytotoxic Activities	Zhu, Q-F.	*Molecules*	2022	https://doi.org/10.3390/molecules27217258
Lactucin, a Bitter Sesquiterpene from *Cichorium intybus*, Inhibits Cancer Cell Proliferation by Downregulating the MAPK and Central Carbon Metabolism Pathway	Imam, K.	*Molecules*	2022	https://doi.org/10.3390/molecules27217358
Anticancer Activity of Mannose-Specific Lectin, BPL2, from Marine Green Alga *Bryopsis plumosa*	Lee, J.	*Marine Drugs*	2022	https://doi.org/10.3390/md20120776
Ethanolic Extract of *Ocimum sanctum Linn.* Inhibits Cell Migration of Human Lung Adenocarcinoma Cells (A549) by Downregulation of Integrin αvβ3, α5β1, and VEGF	Kustiati, U.	*Scientia Pharmaceutica*	2022	https://doi.org/10.3390/scipharm90040069
Libertellenone T, a Novel Compound Isolated from Endolichenic Fungus, Induces G2/M Phase Arrest, Apoptosis, and Autophagy by Activating the ROS/JNK Pathway in Colorectal Cancer Cells	Gamage, C.	*Cancers*	2023	https://doi.org/10.3390/cancers15020489
New Affordable Methods for Large-Scale Isolation of Major Olive Secoiridoids and Systematic Comparative Study of Their Antiproliferative/Cytotoxic Effect on Multiple Cancer Cell Lines of Different Cancer Origins	Papakonstantinou, A.	*IJMS*	2023	https://doi.org/10.3390/ijms24010003
Synthesis and Anti-Proliferative Evaluation of Arctigenin Analogues with C-9′ Derivatisation	Paulin, E.	*IJMS*	2023	https://doi.org/10.3390/ijms24021167
Trichosanthin Promotes Anti-Tumor Immunity through Mediating Chemokines and Granzyme B Secretion in Hepatocellular Carcinoma	Wang, K.	*IJMS*	2023	https://doi.org/10.3390/ijms24021416
Camptothecin Effectively Regulates Germline Differentiation through Bam-Cyclin A Axis in *Drosophila melanogaster*	Zhang, J.	*IJMS*	2023	https://doi.org/10.3390/ijms24021617
JI017 Induces Cell Autophagy and Apoptosis via Elevated Levels of Reactive Oxygen Species in Human Lung Cancer Cells	Ku, J.	*IJMS*	2023	https://doi.org/10.3390/ijms24087528
α-Tocotrienol and Redox-Silent Analogs of Vitamin E Enhances Bortezomib Sensitivity in Solid Cancer Cells through Modulation of NFE2L1	Ishii, K.	*IJMS*	2023	https://doi.org/10.3390/ijms24119382
Modulation of the Endomembrane System by the Anticancer Natural Product Superstolide/ZJ-101	Sanchez, P.	*IJMS*	2023	https://doi.org/10.3390/ijms24119575
Mitochondria-Targeting 1,5-Diazacyclooctane-Spacered Triterpene Rhodamine Conjugates Exhibit Cytotoxicity at Sub-Nanomolar Concentration against Breast Cancer Cells	Heise, N.	*IJMS*	2023	https://doi.org/10.3390/ijms241310695
Phytochemical Analysis and Anticancer Properties of *Drimia maritima* Bulb Extracts on Colorectal Cancer Cells	Al-Abdallat, K.	*Molecules*	2023	https://doi.org/10.3390/molecules28031215
Synthesis of Oleanolic Acid-Dithiocarbamate Conjugates and Evaluation of Their Broad-Spectrum Antitumor Activities	Tang, L.	*Molecules*	2023	https://doi.org/10.3390/molecules28031414
Genistein Inhibits Proliferation and Metastasis in Human Cervical Cancer Cells through the Focal Adhesion Kinase Signaling Pathway: A Network Pharmacology-Based In Vitro Study in HeLa Cells	Chen, T.	*Molecules*	2023	https://doi.org/10.3390/molecules28041919
Anti-Proliferative and Pro-Apoptotic vLMW Fucoidan Formulas Decrease PD-L1 Surface Expression in EBV Latency III and DLBCL Tumoral B-Cells by Decreasing Actin Network	Saliba, J.	*Marine Drugs*	2023	https://doi.org/10.3390/md21020132
A Novel Aldisine Derivative Exhibits Potential Antitumor Effects by Targeting JAK/STAT3 Signaling	Wang, D.-P.	*Marine Drugs*	2023	https://doi.org/10.3390/md21040218
Light-Mediated Transformation of Renieramycins and Semisynthesis of 4′-Pyridinecarbonyl-Substituted Renieramycin-Type Derivatives as Potential Cytotoxic Agents against Non-Small-Cell Lung Cancer Cells	Sinsook, S.	*Marine Drugs*	2023	https://doi.org/10.3390/md21070400
Immuno-Modulatory Effects of Korean Mistletoe in MDA-MB-231 Breast Cancer Cells and THP-1 Macrophages	Lim, W.-T.	*Scientia Pharmaceutica*	2023	https://doi.org/10.3390/scipharm91040048
Molecular Mechanism of Tanshinone against Prostate Cancer	Li, W.	*Molecules*	2022	https://doi.org/10.3390/molecules27175594
Can Natural Products Targeting EMT Serve as the Future Anticancer Therapeutics?	Anwar, S.	*Molecules*	2022	https://doi.org/10.3390/molecules27227668
Natural Products and Small Molecules Targeting Cellular Ceramide Metabolism to Enhance Apoptosis in Cancer Cells	Afrin, F.	*Cancers*	2023	https://doi.org/10.3390/cancers15184645
